# Development of a double-antibody sandwich ELISA for rapid detection of *Bacillus Cereus* in food

**DOI:** 10.1038/srep16092

**Published:** 2016-03-15

**Authors:** Longjiao Zhu, Jing He, Xiaohan Cao, Kunlun Huang, Yunbo Luo, Wentao Xu

**Affiliations:** 1 Laboratory of Food Safety, College of Food Science and Nutritional Engineering, China Agricultural University, Beijing 100083, China; 2 The Supervision, Inspection and Testing Center of Genetically Modified Organisms, Ministry of Agriculture, Beijing, 100083, China

## Abstract

*Bacillus cereus* is increasingly recognized as one of the major causes of food poisoning in the industrialized world. In this paper, we describe a sensitive double-antibody sandwich enzyme-linked immunosorbent assay (ELISA) that was developed for rapid detection of *B. cereus* in food to minimize the risk of contamination. The polyclonal antibody (pAb) and monoclonal antibodies (mAbs) specific to *B. cereus* were generated from rabbit antiserum and mouse ascites, respectively, using the octanoic acid/saturated ammonium sulfate precipitation method and protein A-sepharose columns. IgG-isotype mAbs were specially developed to undergo a novel peripheral multiple sites immunization for rapid gain of hybridomas and a subtractive screen was used to eliminate cross reactivity with closely related species such as *Bacillus thuringiensis*, *B. subtilis, B. licheniformis* and *B. perfringens*. The linear detection range of the method was approximately 1 × 10^4^–2.8 × 10^6^ cells/mL with a detection limit (LOD) of 0.9 × 10^3^ cells/mL. The assay was able to detect *B. cereus* when the samples were prepared in meat with various pathogens. The newly developed analytical method provides a rapid method to sensitively detect *B. cereus* in food specimens.

*Bacillus cereus* is a gram-positive, aerobic-to-facultative, spore-forming, rod-shaped bacterium^1^ and is widely distributed throughout the environment^2^. Hague’s remarkable experiments in the 1950s showed *B. cereus* to be the causative agent of food poisoning^3^. The bacterium causes two distinct forms of gastroenteritis connected to food poisoning, specifically the emetic and the diarrheal types. *B. cereus* food poisoning is underreported as both forms of illness are relatively mild and usually last less than 24 h. However, occasional reports have described a more severe form of the diarrheal type of *B. cereus* food poisoning that has involved hospitalization and even death^4^. *B. cereus* is a common soil saprophyte and is easily spread to many types of food, especially of plant origin, though it is also frequently isolated from meat, eggs and dairy products^4^. There have been several outbreaks in which turkey loaf, sprouts, meat loaf, rice, mashed potatoes, beef stew, egg and apples were implicated^5^,^6^,^7^,^8^,^9^. Due to the increasing incidence of *B. cereus* food-borne illness and the wide spread distribution of *B. cereus* in food, rapid detection methods are required for diagnostic purposes and for the prevention of food contamination and food-borne outbreaks. The minimal level required to provoke both types of diseases was estimated to be approximately >10^5^ colony-forming units (cfu)/g of ingested food. However, there are some reports of emetic syndrome associated with foods containing only 10^3^ cfu/g of food^4^,^10^.

Early studies describing the identification techniques of *B. cereus* are mainly based on the use of selective media and biochemical tests. These methods involve enrichment, plating and specific identification and are laborious, time-consuming and require trained personnel to detect traces of *B. cereus* cells in food^11^. Recently, molecular diagnostic PCR assays have been developed to detect various microbial pathogens, and there are quantitative PCR methods for detecting viable bacteria^12^,^13^, real-time quantitative PCR methods for detecting *Escherichia coli O157: H7*^14^ and a multiplex PCR approach for the simultaneous detection of various food-born pathogen^15^. However, conventional PCR requires post-PCR analysis by gel electrophoresis, which bears the risk of false-positive results due to laboratory contamination^16^. As the detection target of PCR-based methods have mainly focus on specific gene sequences, these methods often fail to eliminate the complicated protocol of cell disruption and nucleic acid extraction; thus, to some extent these methods do not support convenient, rapid and real-time detection of *B. cereus*^17^.

Enzyme-linked immunosorbent assay (ELISA) is a specific, sensitive, and convenient method for measuring macromolecular protein, polysaccharide and bacteria. The method is precise and reproducible and employs stable reagents and inexpensive equipment. Therefore, ELISA is applicable to routine detection of *B. cereus* in clinical and research laboratories. Thus, this method is attractive for on-site applications. Some immunological assays have been made commercially available, such as the *B. cereus* Enterotoxin Test Kit, although commercial kits are not yet available for whole cells of *B. cereus* strains^1^.

Based on the facts in this study, we use the *B. cereus* whole cells as immunogens to generate rabbit polyclonal and mouse monoclonal antibodies, which can recognize certain surface components of *B. cereus*. These antibodies were then used to develop a sandwich ELISA for fast, direct and convenient measurement of *B. cereus* contamination of food. To the best of our knowledge, this is the first sandwich ELISA reported for direct and species-specific detection of *B. cereus* cells.

## Materials and Methods

### Reagents and animals

*B. cereus ATCC11778* and related species strains analyzed in this study were stored at −80 °C. Incomplete Freund’s adjuvant, 50% polyethylene glycol-1450 (PEG_1450_), paraformaldehyde, RPMI1640, fetal calf serum (FCS), hypoxanthine/aminopterin/thymidine (HAT), hypoxanthine/thymidine (HT), methyl cellulose, 3, 3, 5, 5-tetramethylbenzidine (TMB), and mouse monoclonal antibody ISO2-1 kits were purchased from Sigma-Aldrich (Shanghai, China). Goat anti-mouse immunoglobulin horseradish peroxidase conjugate, goat anti-rabbit immunoglobulin horseradish peroxidase conjugate (IgG–HRP) were purchased from Univ-bio (Shanghai, china) and streptavidin-horseradish peroxidase conjugate were purchased from Invitrogen (Shanghai, china). The Sephadex G-25 column and protein A-sepharose columns were purchased from General Electric Company (GE) (Shanghai, China). All other reagents were of analytical grade. The ELISA was conducted in EIA 1 × 8 Stripwell^TM^ Plates, No Lid, 42592 (Costar, USA) using a 12-channel pipette (50 ~ 300 μL, Thermo Lab Systems Co. Ltd., Shanghai, China). The absorbance at 450 nm was scanned in each well with a Varioskan Flash (Thermo, USA). BABL/c murine myeloma cells SP2/0 were conserved by our laboratory. BABL/c mice were obtained from Charles River Company (Beijing, China). The amounts (cells/mL) of *B. cereus* were counted in a Petrof Hausser bacteria chamber.

### Ethics Statement

All animal procedures involving the care and use of animals were practiced in accordance with the ethics regulations of science research in the Institute of the Supervision, Inspection and Testing Center of Genetically Modified Organisms, Ministry of Agriculture (Beijing, China) and were approved by the Animal Experimental Welfare & Ethical Inspection Committee (No. 100034).

### Buffers and antigen preparation

The ELISA buffers used regularly include (a) coating buffer, 50 mM carbonate buffer (pH 9.6):1.59 g Na_2_CO_3_, 2.93 g NaHCO_3_ in 1 L distilled water; (b) dilution buffer, 10 mM PBS (pH = 7.4): 2.9 g Na_2_HPO_4_·12H_2_O, 8 g NaCl, 0.2 g KCl, and 0.2 g KH_2_PO_4_ in 1 L distilled water; (c) washing buffer (PBST), 10 mM PBS (pH 7.4) containing 0.05% (v/v) Tween 20; (d) double blocking buffer, 10 mM PBS (pH 7.4) containing 0.05% (m/v) BSA and 0.05% (m/v) skim milk powder solution; (e) TMB solution, 1 mL sodium citrate buffer (pH 3.6) containing10 μL 10 mg/mL TMB and 1 μL 30% (v/v) H_2_O_2_; and (f) stop solution: 2 M H_2_SO_4_.

The antigen preparation involved (a) whole-cell lysates antigen prepared in 50 mM Tris-HCl, 10 mM EDTA, 100 mM NaCl, pH = 8.0, incubated for 30 min at 37 °C, followed by 150 W ultrasonication (10 s, 10 s, 90s cycle 40 times); (b) cell-surface extract antigen: antigen b1, *B. cereus* was in 5 mM Tris hydrochloride (TRISE), 1% (wt/vol) SDS, and 5 mM 2-mercaptoethanol, pH=9.8, heated to 70 °C for 30 min^18^,^19^; antigen b2, *B. cereus* was in 5 mM Tris hydrochloride (TRISE) and 1% (wt/vol) SDS, pH = 8.0, incubated at room temperature for 30 min^18^,^19^ (c) antigen boiling: antigen was boiled in water for 10 min (d) formaldehyde fixation of antigen: antigen was fixed in 4% formaldehyde for 20 min at room temperature; (e) paraformaldehyde fixation of antigen: antigen was fixed in 4% paraformaldehyde for 20 min at room temperature.

### Bacterial strains and whole-cell immunogen preparation

For immunogen preparation, bacteria were cultured in 20 mL of Luria-Bertani (LB) medium at 37 °C for 12 ~ 14 h. Bacteria cells were harvested by the cultures centrifuge for 10 min at 10,000 rpm and remove the supernatant fluid. The cell pellets were washed three times with sterile saline (0.9% NaCl) and fixed for 20 min at room temperature in 4% paraformaldehyde^20^. The fixed whole cells were washed three time in sterile saline, centrifuged (10,000 rpm, 20 min), and then suspended in sterile saline for injection. Bacterial concentrations of the final suspension were determined in a Petrof Hausser bacteria chamber.

### Whole-cell immunization protocols of animals

Two protocols for immunization were carried out. The first immunization procedure was described by Caterson^21^ with slight modifications. Each 8-week-old male BALB/c mouse was immunized with whole cell immunogen at six sites over a 13-day immunization protocol. The amounts inoculated were ~10^8^, ~10^7^, ~10^5^ ~10^4^, ~10^4^, and ~10^4^ cells on days 1, 3, 6, 8, 10 and 13, respectively. At each site, the immunogen was dissolved in 100 μL of sterile phosphate buffered saline (PBS) without any adjuvant. The *B. cereus* whole cell immunogen was injected intramuscularly at bilateral sites (calf, groin and axillary) adjacent to lymph nodes. Blood samples were collected from the mouse tail on the nineteenth day. The titers were determined by enzyme-linked immunosorbent assay (ELISA).

The second protocol followed conventional subcutaneous (s.c.) injection with slight modifications^22^. The immunogen was also adjuvant-free and delivered to six sites on the nape of the neck at weeks 1, 4, 6 and 8. The immunizing dose of *B. cereus* was a fixed level of ~10^8^ cells. Three days prior to cell fusion, the mice were boosted with ~10^9^ cells by intraperitoneal (i.p.) injection. The 12-week-old female adult rabbits were also immunized according to the second protocol. However, the dose of immunization for rabbits was ten-fold higher than that used with mouse. Blood samples were collected from the mouse tail or rabbit ear vein on the seventh day following each boost. The titers were determined by enzyme-linked immunosorbent assay (ELISA).

### Rabbit polyclonal antibody production

Seven days after the final injection, the rabbit was anesthetized by intraperitoneal injection of 10% chloral hydrate to collected whole blood and antiserum. Immunoglobulin G (IgG) was purified from the antiserum using octanoic acid-saturated ammonium sulfate precipitation^23^ and protein A-sepharose columns^24^. Then, the purified antibody was desalinated over a Sephadex G-25 column. The purified polyclonal antibody was stored at −20 °C. The antibody titers were assayed by indirect enzyme-linked immunosorbent assay (ELISA).

### Production of mAbs

#### Fusion protocol

Seven days after the final booster immunization, a single-cell suspension was prepared aseptically in RPMI-1640 medium from the inguinal, mesenteric lymph nodes and spleen, which were harvested from sacrificed animals. The splenocyte and lymphocyte suspensions (~5.9 × 10^7^ cells with 80% viability, as determined by trypan blue staining) were prepared with a tissue grinder and 200 mesh sieve cells. The cell suspensions were mixed with murine myeloma cells SP2/0 (0.8 × 10^7^ cells) at a ratio of approximately 5–10:1. The mixed cell suspension was centrifuged and the supernatant was removed completely. The combined lymphocyte/splenocyte suspension and sp2/0 cells were fused using a modification of the method described by Galfre G *et al.*^25^. One milliliter of 50% polyethylene glycol 1450 (PEG_1450_) was added drop wise to the mixed cell pellet in a 50 mL centrifuge tube over 90 s. The PEG-cell mixture was gently stirred while PEG_1450_ was added and then was allowed to incubate for 1 min. The fusion was stopped by drop-wise addition of 50 mL of RPMI-1640 to the mixture at 1 mL in 60 s; 2 mL in 30 s; 3 mL in 30 s, and so forth until all of the RPMI-1640 was added. The temperature was maintained at 37 °C throughout the addition. The cell suspension was then centrifuged for 7 min at 1500 rpm; afterward, the pelleted cells were resuspended gently in 20 mL of HAT complete liquid medium. The fusion suspension was gently mixed with 80 mL of semi-solid complete medium containing 1 g methyl cellulose. Then, 1.5 mL fusion cell suspension was distributed into 6-well plates and incubated in 5% CO_2_ at 37 °C.

### Hybridoma subtractive screening and mAb generation

After still culture of cells over 7–12 days, the semi-solid selective complete medium displayed many white dots suggestive of monoclonal hybridoma, which could be seen by the naked eye. These white dots were transferred into four 96-well culture plates to expand and undergo a subtractive screen for gaining hybridoma cell lines which secreting anti-*B. cereus* antibody. Anti-*B. cereus* antibody-secreting hybridoma cell lines were injected into the abdomens of 7 week old BALB/c mice (2 × 10^6^ cells for each mouse) at seventh day after 400 μl Freund’s incomplete adjuvant was preinjected^26^. The ascites could be obtained by extraction with a 50-mL syringe approximately seven days later. The mAbs were purified from ascites using the octanoic acid/saturated ammonium sulfate precipitation method and were subsequently purified by protein A-sepharose columns^24^. Purified mAbs were immediately neutralized with 1.0 M Tris, pH = 9.0, desalinated over a Sephadex G-25 column, and stored at −20 °C.

### Characterization of antibody

Determination of antibody titers was conducted via indirect ELISA (~10^9^ cells in carbonate-coating buffer). Appropriate dilutions (at least eight serial dilutions) of the purified antibody were made for the experiments. The antibody titer was defined as the reciprocal of the highest antibody dilution that gave an absorbance greater than or equal to 2.1 fold of the background absorbance of PBS (the negative control) in the first dilution^27^.

Subclass of the monoclonal antibodies was performed by a direct ELISA using a commercially available ISO2-1 kit from Sigma.

The affinities of the antibodies were determined by a non-competitive enzyme immunoassay, as described by Beatty, Barbara and William^28^,^17^.

### The boiled bacteria antigen and cell-surface extract antigen were applied to a SDS-polyacrylamide gel

The *B. cereus* cells were boiled for 10 min then centrifuged at 10,000 rpm for 30 min to separate the boiled sediment (c1) and supernatant (c2) and applied to a SDS-polyacrylamide gel. Following electrophoresis, the proteins were transferred onto a nitrocellulose membrane (Millipore). The membrane was washed with TBS-T three times for 15 min and then blocked with 5% BSA for 1 h at room temperature, followed by incubation with mAb (1:1000) for 3 h. After applying the mAb solution and washing with TBS-T, the anti-mouse immunoglobulin horseradish peroxidase conjugate (IgG-HRP, 1:1000 dilution) was added and incubated for 1 h at room temperature. The membrane was then washed and incubated with BCIP/NBT (Amresco, Beijing) solution in a dark room for 15 min^29^.

The immunoassay that was used to test for potential steric competition^30^ of mAbs 2C2 and 2D2 was performed as a double-antibody capture assay. Briefly, microplates were coated with 10^9^ cells/mL suspensions of *B. cereus* in a volume of 100 μL and were blocked with 0.05% skim milk powder for 1.5 h before use. Sequential dilutions of unlabeled mAbs (1:500, 1:1,000, 1:2,000, 1:4,000, 1:8,000, and 1:16,000) were transferred to the coated and blocked plate at 50 μL/well. Plates were incubated for 1 h at 37 °C. 50 μL biotinylated mAb (diluted 1:500) was added at the same volumes without removing the content of the wells. The biotinylated mAb was diluted 1:1,000 and one of each of the six sequential dilutions of unlabeled mAbs (from 1:1,000 to 1:32,000) was to the final reaction mixture in a volume of 100 μL. The mixture was incubated for 1h at 37 °C. Plates were washed three times and incubated for 30 min with streptavidin-horseradish peroxidase conjugate followed by addition of a substrate for HRP (TMB, H_2_O_2_). The stain was developed after 15 min, then stopped by 2M H_2_SO_4_ and read at a wavelength of 450 nm.

### Detection of *B. cereus* by sandwich ELISA

Different concentrations of *B. cereus* whole-cell suspensions ranging from approximately 10^4^ to 10^6^ cells/mL were determined by a sandwich ELISA according to the following protocol: (1) the micro wells of the ELISA plate were coated with 100 μL of capture antibody (pAb) at 18 μg/mL in coating buffer at 4 °C overnight; (2) three washes with PBS-T were performed to remove unbound antibody and each well was blocked with 200 μL of 0.05% skim milk powder and incubated at 37 °C for 90 min; (3) the washing step was repeated and100 μL of ten-fold serial diluted samples (*B. cereus*) were added to each well at 37 °C for 40 min; (4) washes were repeated and 100 μL of the monoclonal antibody 2D2 at a 1:1000 dilution in PBS was added to each well and incubated at 37 °C for 30 min; (5) washing was repeated and100 μL goat anti-mouse IgG horseradish peroxidase conjugate (at a 1:5000 dilution in PBS) was then added to each well and was incubated at 37 °C for 30 min; (6) the plate was then washed six times and 100 μL of TMB solution was added to each well; (7) plates were incubated at 37 °C for 15 min; the reaction was then terminated by the addition of 50 μL of 2 M H_2_SO_4_ and the absorbance values at 450 nm were measured.

### Preparation of food samples contaminated by various pathogens

Comminuted meat was chosen as a food sample for detection of *B. cereus* under various bacterial contaminations. Twenty-five grams of comminuted meat and 10^5^ cells of *Bacillus thuringiensis*, *Bacillus perfringens, Bacillus subtilis, Bacillus licheniformis*, *Lactobacillus casei, Escherichia coli*, *Listeria monocytogenes* and *Salmonella* (characteristics of the 8 strains are listed in Table 1) were mixed with 225 mL of 0.02% Tween 20-buffered peptone water (Tween20-BPW) and homogenized for 1 min. The Tween 20 in BPW is used to emulsify the fat in the samples. Approximately 220 mL the homogenate filtrate was removed and centrifuged at 8000 rpm for 5 min at room temperature. The supernatant was transferred to a new tube and centrifuged at 10, 000 rpm for 5 min at room temperature. The final pellet was suspended in 1.5 mL of 10 mM PBS for detection.

## Results and Discussion

### Immunization strategy and results

**Figure 1:**
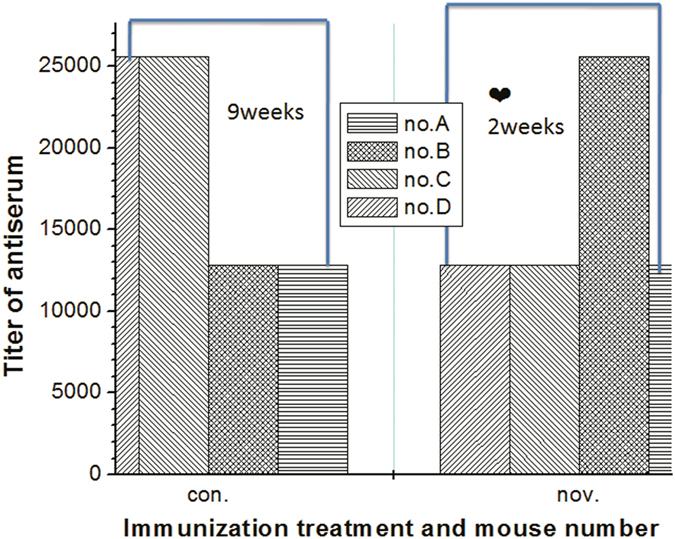
Comparison of the antisera titer and immunization period of the immunized mice used in the con (conventional) and nov (novel) immunization procedures.

In this paper two protocols were used for animal immunization. The first injection protocol involved immunization at multiple peripheral sites, which provides an opportunity to rapidly produce affinity-matured (IgG) antibody-secreting immune cell lines within 13 days of the primary immunization. The second immunization technique used followed the conventional procedure and requires approximately 3 months. The first procedure significantly saves preparation time of mAb development and results of similar titers. This novel immunization protocol is based on that B lymphocytes may have rapid affinity maturation within secondary lymphatic tissues and an abbreviated immunization regimen. This finding is also supported by a description of T cell-dependent B cell responses following antigenic challenge^31^. Figure 1 shows the immunization results produced using the conventional and novel protocols, which are described in detail in the whole-cell immunization protocols of animals section. Both protocols induced optimal results. However, the conventional injections required a longer immunization period of 9 weeks while the novel injection protocol requires a relatively short immunization period of 2 weeks. In this paper, all of the injections are free with adjuvant because the paraformaldehyde-fixed whole-cell immunogen was water-insoluble particle antigens which can slow self-release without adjuvant. After the injections were completed, popliteal lymph nodes and the spleen were markedly tumescent.

### Hybridoma subtractive screening

**Figure 2:**
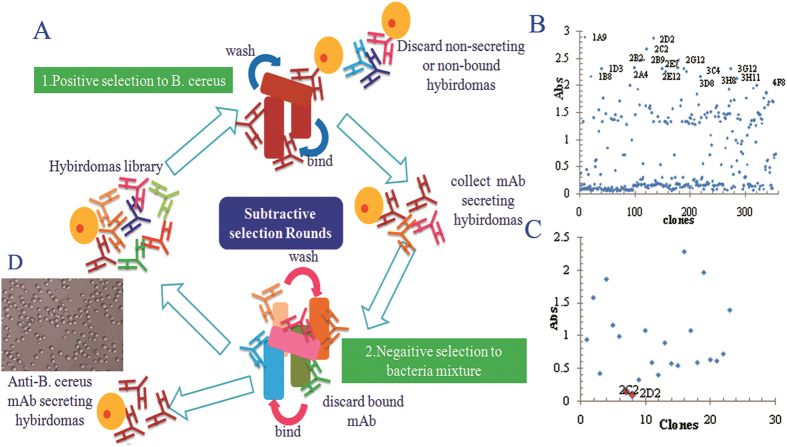
(**A**) The principle of the subtractive screen. (**B**) Twenty-three hybridomas were generated against *B. cereus* by the eighth positive screen. (**C**) Two non-cross-reactive mAb-secreting hybridomas were generated by the fourth negative selection. (**D**) The morphology of hybridomas observed under the microscope. All of the selections were conducted by the indirect ELISA.

**Figure 3:**
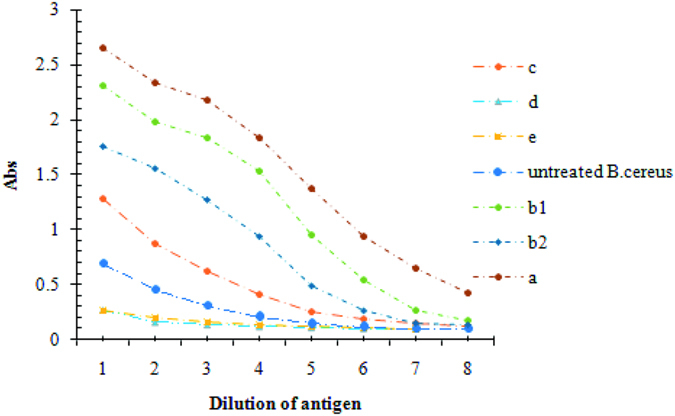
The coating capacity of different treatments of *B. cereus* antigen. (**a**) whole-cell lysates antigen prepared in 50 mM Tris-HCl, 10 mM EDTA, 100 mM NaCl, pH = 8.0, incubated for 30 min at 37 °C, followed by 150 W ultrasonication (10s, 10s, 90s cycle 40 times); (**b**) cell-surface extract antigen: antigen b1, *B. cereus* was in 5 mM Tris hydrochloride (TRISE), 1% (wt/vol) SDS, and 5 mM 2-mercaptoethanol, pH = 9.8, heated to 70 °C for 30 min; antigen b2, *B. cereus* was in 5 mM Tris hydrochloride (TRISE) and 1% (wt/vol) SDS, pH = 8.0, incubated at room temperature for 30 min (**c**) antigen boiling: antigen was boiled in water for 10 min; (**d**) formaldehyde fixation of antigen: antigen was fixed in 4% formaldehyde for 20 min at room temperature; (**e**) paraformaldehyde fixation of antigen: antigen was fixed in 4% paraformaldehyde for 20 min at room temperature.

The hybridomas were screened using an indirect ELISA. Traditional screening techniques for the production of monoclonal antibodies usually depend on the generation of monoclonal antibodies against immunodominant molecules. Some extent of cross-reactivity was expected in the hybridomas due to conserved immunodominant antigens shared by closely related bacteria. To enhance the production of monoclonal antibodies against rare or special immunodominant antigens on *B. cereus*, a subtractive screening technique was first employed. The principle of the subtractive selection is illustrated in Fig. 2A. *Bacillus thuringiensis*, *Bacillus subtilis, Bacillus licheniformis* and *Bacillus perfringens* were incorporated into the negative step of the selection to ensure a high specificity of the generated mAb for *B. cereus*. We also added double blocking buffer (0.05% BSA and 0.05% skim milk powder) in the positive and negative selection steps to eliminate nonspecific binding and increase the stringency of the mAb selection. It should be noted that the 4% paraformaldehyde-fixed bacteria cells show extremely low coating on microwells for indirect ELISA, and the use of boiled bacteria antigen (antigen(c) in the buffers and antigen preparation section), whole-cell lysates antigen (antigen(a)) and cell surface antigen (antigen(b1,b2)) demonstrated the robust coat capacity for indirect ELISA screening (Fig. 3). This result may be because water boiling, fragmentation and surface extraction can release many water-soluble surface components of *B. cereus*. However, the paraformaldehyde–fixed bacteria may form a hydrophobic layer to hinder the cross linking of water-soluble surface components of *B. cereus* with the microporous polyethylene plate. The formalin-treated cells also exhibit a low coating capacity in accordance with the paraformaldehyde-fixed cells. In this paper, we choose antigen c as coat antigen due to it showed moderate coating capacity with intact cell. Finally, we selected two highly positive hybridomas 2C2 and 2D2 (Fig. 2D) from the 355 hybridomas in the eighth round of positive selection and fourth round of negative screening (Fig. 2B,C).

### Characterization of mAb

**Figure 4:**
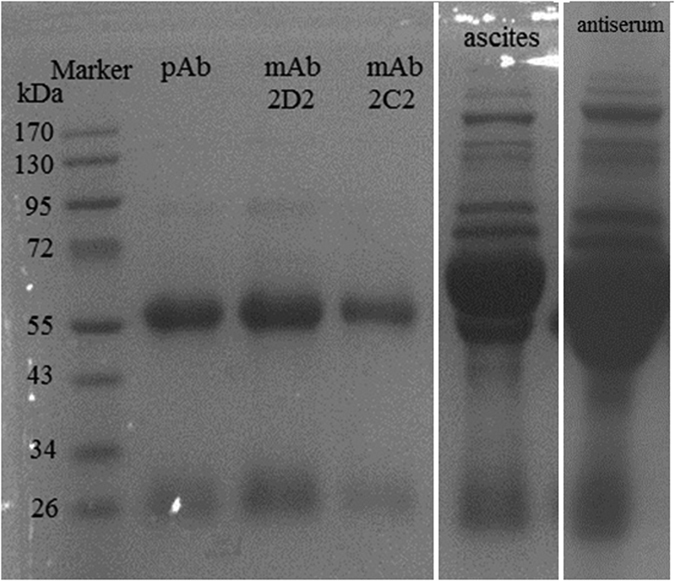
Gel electrophoresis of the purified polyclonal antibody (pAb); the monoclonal antibodies 2D2 (mAb 2D2) and mAb 2C2; the unpurified ascites and antiserum.

The hybridoma cell lines which secrete IgG mAb were expanded and injected into the abdomens of 7-week-old BALB/c mice at 7 days after Freund’s Incomplete Adjuvant was injected. The mAbs and pAb were purified from ascites and rabbit antiserum using the octanoic acid/saturated ammonium sulfate precipitation method and protein A-sepharose column purification. The SDS-PAGE results demonstrate that the purified antibodies have the characteristic 60-kDa (heavy chain) and 25-kDa (light chain) bands and extraneous proteins from the ascites and antiserum have been entirely eliminated (Fig. 4). The final purity of the IgG was calculated after densitometry of the stained electrophoretogram by BandScan software. The purity of the antibody was 98%. The titer of the purified mAb 2C2 and 2D2 was 12,800 and 25,600, respectively. The average affinity was 8.62 × 10^9^ L/mol and 5.20 × 10^9^ L/mol calculated by the methods developed by Beatty, Barbara and William^17^,^28^. The two monoclonal antibodies had the same isotype determined as IgG_1_.

### Western blotting and steric competition

**Figure 5:**
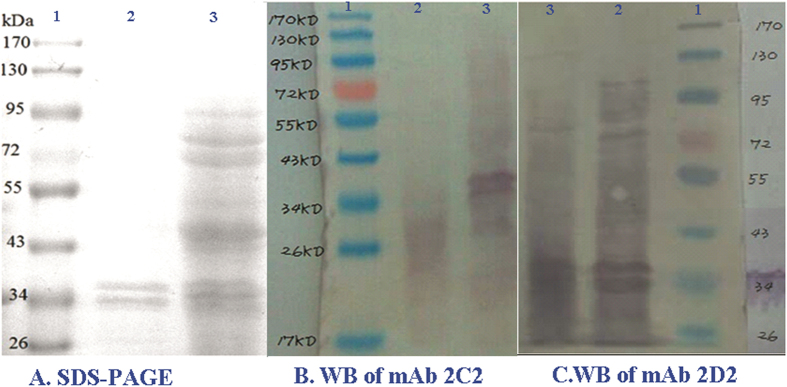
SDS-PAGE and Western blotting analysis. (**A**) The SDS-PAGE profile of *B. cereus* cells (Lane 2) and the cell-surface extract antigen b1 (Lane 3), Lane 1: Protein Marker. (**B**) The WB profiles of mAb 2C2. Lane 2: *B. cereus* cells, Lane 3: cell-surface extract antigen b1, Lane 1: Protein Marker. (**C**) The WB profiles of mAb 2D2. Lane 3: *B. cereus* cells, Lane 2: cell-surface extract antigen b1, Lane 1: Protein Marker.

To determine the nature of the antigens recognized by the purified mAb, boiled bacteria antigen and cell-surface extract antigen were electrophoresed on SDS-PAGE gels and probed by Western blotting. The results indicate that the mAb 2D2 recognizes a 34 kDa component of *B. cereus*. The mAb 2C2 recognizes a target component of 34 kDa (Fig. 5). This result suggests that the hybridoma cell lines 2C2 and 2D2 produced antibodies against same antigen. The steric competition experiments were set up in the format of an antibody capture assay. The two mAbs both showed a “self-competition” of 100% and a “cross-competition” of 90%. The evident competition between the two mAbs indicated that the recognized epitopes of 2C2 and 2D2 were overlapped, which is consisted with the western blot analysis.

Protein EA1 is an abundant, highly antigenic, surface layer protein of *Bacillus anthracis* vegetative cells which is an affiliated bacterium of *B. cereus* (The EA1 of *B. cereus* was homology to *B. anthracis*). Wang group prepared three mAbs that recognized the surfaces of the intact vegetative cells^17^. This group also validated that the 91–93 kDa protein EA1 is the high-affinity target protein of the three mAbs. It should be noted that the western blotting results demonstrated the success of the subtractive screening strategy in avoiding the surface component of closely relative bacteria.

### Standard curve of the sandwich ELISA

**Figure 6:**
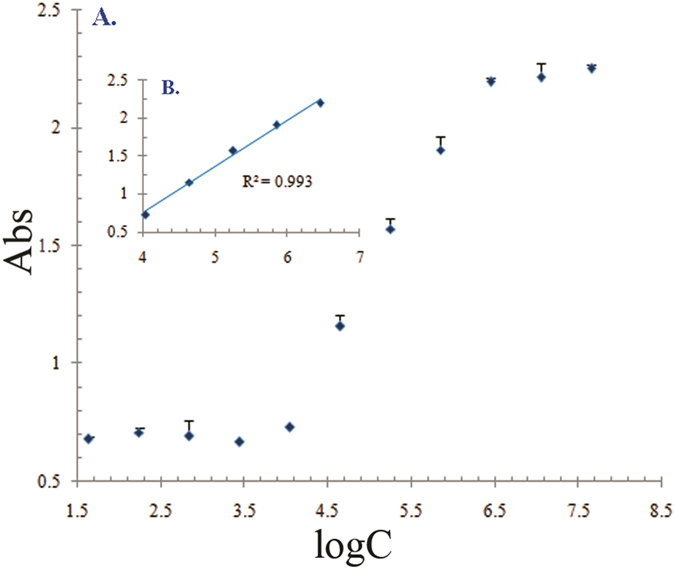
The double-antibody sandwich ELISA for the determination of *B. cereus* using rabbit polyclonal antibody (as capture antibody, 18 μg/mL) and monoclonal antibody 2D2 (as detecting antibody, 1:1000). ‘A’ is standard curve of sandwich ELISA. ‘B’ is linear detection line conserved from ‘A’, linear regression shows a working range from 1 × 10^4^ to 2.8 × 10^6^ cells/mL (r^2^ = 0.993), as in the top left insert. X axle: the absorbance of microwell at 450 nm; Y axle: the log Kow of concentration of *B. cereus.*

The *B. cereus* whole-cell antigen with concentrations ranging from 1 × 10^4^ to 1 × 10^9^ cells/mL was used to construct a standard curve that was fit to the equation y = 0.612x - 1.698. The working range of the assay was defined as the part of the curve with a linear coefficient of r^2^ > 0.99. The linear range included concentrations of 1 × 10^4^ to 2.8 × 10^6^ cells/mL with a detection limit of 0.9 × 10^3^ cells/mL (Fig. 6).

The limit of detection (LOD) was calculated by the standard formula, which is shown below and is further defined in the literature^32^,^33^,^34^:


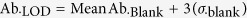


where Ab._LOD_ is the optical density corresponding to the LOD, Ab._Blank_ is the optical density of the blank, and σ_.Blank_ is the standard deviation of the blank from all the repeats.

### Recovery studies

To evaluate the specificity and utility of the double-antibody-based sandwich ELISA, comminuted meat was spiked with various pathogens and subjected to sandwich ELISA analysis. Recoveries of *B. cereus* from spiked food sample ranged from 94.9% to 98.4% with CV (coefficient of variation) values ranging from 1.3% to 7.2%. These results demonstrate that the food environments in which *B. cereus* may be found do not interfere with the assay. Furthermore, other closely related bacteria did not influence the analysis results due to the successful application of the subtractive screen that significantly eliminated cross-reactivity.

## Conclusions

In this paper, we first used a rapid immunization protocol that injected antigen at multiple peripheral sites to shorten the production cycle of mAb. Second, the subtractive screen largely enhanced the specificities of the generated mAbs, which ensured the specificity of the sandwich ELISA. Third, during selection we used double sealants to reduce the non-specific adsorption and to guarantee the fidelity of the isolated mAb. Fourth, the preparation of soluble antigen plays a key role in the enhancement of the coating capacity for indirect ELISA, which facilitates successful subtractive selection. Finally, we used the polyclonal antibody as a capture antibody to offer an optimal trap rate of *B. cereus,* which enhanced the sensitivity of the sandwich ELISA because pAbs can identify more epitopes than mAbs, thus offering more capture opportunities.

In conclusion, highly specific mAbs against *B. cereus* can be rapidly and efficiently produced in mice by the peripheral multiple sites immunization and subtractive screening technique. A double-antibody sandwich ELISA using the mAb and rabbit polyclonal antibody was developed for the detection of *B. cereus*. Based on the assay results, the *B. cereus* can be directly detected at concentrations as low as 0.9 × 10^3^ cells/mL in phosphate buffered saline. The recoveries of *B. cereus* from meat samples ranged from 94.9 to 98.4%. These results suggest that the sandwich ELISA represents a good tool for *B. cereus* detection in food specimens. This strategy can also be used for rapid assay development directed against various other food-borne pathogens and for the screening of microorganism risk factors in food. The procedures enable the application of particular biosensor/diagnostic methods.

## Additional Information

**How to cite this article**: Zhu, L. *et al.* Development of a double-antibody sandwich ELISA for rapid detection of *Bacillus Cereus* in food. *Sci. Rep.* **6**, 16092; doi: 10.1038/srep16092 (2016).

## Figures and Tables

**Table 1:** The bacterial strains used in this paper.

Strain	Number	Source
*Escherichia coli BL21*		stored in our laboratory
*Salmonella*	ATCC 11778	stored in our laboratory
*L. monocytogenes*	CMCC 55004	China Institute of Veterinary Drug Control
*Lactobacillus casei*		Professor Tian, Agriculture University of Hebei
*Bacillus subtilis*		stored in our laboratory
*Bacillus thuringiensis*		stored in our laboratory
*Bacillus licheniformis*	CGMCC 1.106	Institute of Microbiology, Chinese Academy of Sciences
*Bacillus perfringens*		stored in our laboratory
